# Catastrophic health care expenditure in Myanmar: policy implications in leading progress towards universal health coverage

**DOI:** 10.1186/s12939-019-1018-y

**Published:** 2019-07-30

**Authors:** Chaw-Yin Myint, Milena Pavlova, Wim Groot

**Affiliations:** 1 Department of Health Services Research, CAPHRI, Maastricht University Medical Center, Faculty of Health, Medicine and Life Sciences, Maastricht University, P.O. Box 616, 6200 MD Maastricht, The Netherlands; 2 Water, Research and Training Center (WRTC), Yangon, Myanmar; 3 Top Institute Evidence-Based Education Research (TIER), Maastricht University, Maastricht, The Netherlands

## Abstract

**Background:**

Around the world, millions of people are impoverished due to health care spending. The highest catastrophic health expenditures are found in countries in transition. Our study analyzes the extent of financial protection by estimating the incidence of catastrophic health care expenditure in Myanmar and its association with sociodemographic factors.

**Methods:**

We performed a secondary analysis of data from the household surveys conducted by the Livelihoods and Food Security Trust Fund (LIFT) in 2013 and 2015 in Myanmar. To estimate the magnitude of catastrophic health care expenditure, we applied the definition of catastrophic payment proposed by the World Health Organization (WHO); a household’s out-of-pocket payment for health care is considered catastrophic if it exceeds 40% of the household capacity to pay. We also examined the changes in catastrophic payments at three different threshold levels (20, 30, 40%) with one equation allowing for a negative capacity to pay (modified WHO approach) and another equation with adjusted negative capacity to pay (standard WHO approach).

**Results:**

In 2013, the incidence of catastrophic expenditure was 21, 13, 7% (standard WHO approach) and 48, 43, 41% (modified WHO approach) at the 20, 30, 40% threshold level respectively, while in 2015, these estimates were 18, 8, 6% (standard WHO approach) and 47, 41, 39% (modified WHO approach) respectively. Geographical location, gender of the household head, total number of household members, number of children under 5, and number of disabled persons in the household were statistically significantly associated with catastrophic health care expenditures in both studied years 2013 and 2015. Education of household head was statistically significantly associated with catastrophic health expenditure in 2013. We found that the incidence of catastrophic expenditures varied by the approach used to estimate expenditures.

**Conclusions:**

Although the level of catastrophic health care expenditure varies depending on the approach and threshold used, the problem of catastrophic expenditures in Myanmar cannot be denied. The government of Myanmar needs to scale up the current Social Security Scheme (SSS) or establish a new financial protection mechanism for the population. Vulnerable groups, such as households with a household head with a low-level of education, households with children under the age of 5 years or disabled persons, and low-income households should be prioritized by policymakers to improve access to essential health care.

**Electronic supplementary material:**

The online version of this article (10.1186/s12939-019-1018-y) contains supplementary material, which is available to authorized users.

## Introduction

Around the world, millions of people are impoverished due to health care spending or have to spend catastrophic amounts of money for health care. Catastrophic payments impede access to health care. Catastrophic expenditure can occur in every country at all stages of development, even in countries with well-developed financial risk protection mechanisms, e.g., in Australia, the lowest income group had a 15 times higher chance of having catastrophic health expenditure compared to the highest income group [1, 2]. However, the highest catastrophic health expenditures are found in countries in transition. According to a study published in 2003, the proportion of households facing catastrophic health expenditure varies across countries, ranging from 0.01 to 11.4% [3]. A more recent study on 99 countries shows that the global incidence of catastrophic spending at the 10% threshold of household consumption has slightly increased from 9.7% in 2000 to 11.4% in 2005 and to 11.7% in 2010 [4].

In Myanmar, out-of-pocket payments (OOPPs) are the main source of health care financing, representing 74% of the total health expenditures in 2015. This percentage ranks Myanmar as the country with the highest OOPPs for health care among the Association of Southeast Asian Nations (ASEAN) [5]. Xu, Evans, et al. 2003 find that the higher the OOPP contribution to the total health expenditure is, the higher the chance of catastrophic payments is [3]. OOPPs for using health services can impact equity in accessing health care as well as the economic status at the population. In particular, some household members may choose to forego health care use if the household is not able to make the related OOPPs. Alternatively, households may experience catastrophic spending if they choose to seek services beyond their ability to pay. At the same time, wealthier households may not be affected by this as they are more able to afford OOPPs [3]. Generally, countries with a prepayment system or social protection system provide better access to care and are less burdened by catastrophic health care expenditures [6, 7]. However, such protection mechanisms are largely missing in Myanmar. The Social Security Scheme (SSS) in Myanmar was established in 1957, but at present, it protects only 1% of the population. The SSS is not yet ready to expand its coverage because of low capacity of the supply-side.

There are only a small number of studies that have estimated catastrophic health care expenditure in Myanmar. The studies that have investigated this expenditure find that catastrophic payments range between 10 and 40%, depending on the method applied [8–12]. However, these studies have been unable to disaggregate the OOPPs amount for specific health care services, such as pharmaceuticals, medical products, outpatient care, dental, travel, and inpatient care.

Our study explores not only the extent of financial protection conferred by estimating the incidence of catastrophic health care expenditure in Myanmar and its association with sociodemographic factors but also highlights the share of OOPPs that is spent on each type of service such as pharmaceuticals, outpatient care, and others. We performed a secondary analysis of data from the household surveys conducted by the Livelihoods and Food Security Trust Fund (LIFT) in 2013 and 2015 in Myanmar. The LIFT was initiated in 2010 to assist the poor with food availability and income. The LIFT program provides inputs for agriculture (e.g., seed, credit, and equipment) and non-agricultural livelihood (e.g., capital investments, credit, training, technical assistance, and marketing support), as well as advice on natural resource management (e.g., community forestry and mangrove rehabilitation and embankments against flooding) and support to develop effective social protection measures, especially for the chronically poor. The LIFT household survey was conducted among all population groups (i.e., those covered and not covered by the LIFT) in the country’s main agro-ecological zones: Hilly zone, Dry zone, and Delta zone.

Our study is important because it examines the level of financial protection, which is one of the monitoring indicators for health financing arrangements in a country. We use regionally representative data provided by the LIFT survey [13]. Evidence on the association between sociodemographic factors and catastrophic health care expenditures highlights vulnerable groups who should be the priority in social protection policy in Myanmar. We also use two different approaches to check the robustness of our findings, which may be of interest for countries conducting similar studies. Thus, our results are relevant for Myanmar as well as for other low-income countries dealing with high OOPPs.

## Data and methods

### Data sources

The LIFT household survey was conducted as part of the evaluation of LIFT activities described above. Three survey waves were carried out in 2011, 2013, and 2015 respectively in the main agro-ecological zones: Hilly zone, Dry zone, and Delta zone. These zones cover 76 townships in 8 of 15 states/regions in Myanmar. However, data on expenditures were only collected in 2013 and 2015, so data from those years were only used in our analysis. The surveys were carried out among 200 villages. Although the survey was conducted among 16 households in each village, data on expenditures were only collected among 5 households in each village. Overall, the sampling procedure for the two years was comparable. The total sample for the expenditure part of the survey was 1000 in 2013 and 1165 in 2015 [14].

The expenditure survey questions were adapted from the 2009–2010 Integrated Household Living Conditions Assessment survey conducted in Myanmar, which was based on the World Bank’s Living Standards Measurement Study surveys. The questions were adapted to the Myanmar context by replacing local foods and other goods consumed in the country. We did not participate in the design of the survey and in the data collection process. The data from the expenditure module that we used for our analysis were anonymized before being provided to us.

The expenditure dataset is divided into six sections:

- Food consumption expenditures over the last 7 days at home: pulses, beans, nuts, and seeds; meat, dairy, eggs; fish and other seafood; roots and tubers; vegetables; fruits; spices and condiments; other food products except tobacco and alcohol.
- Other food consumption expenditures during the last 7 days: alcoholic beverages consumed at home or outside of home; food and beverages taken outside home
- Food consumption expenditures during the last 30 days: rice and cereals; oil and fats; milk products; other food items
- Nonfood consumption expenditures during the last 30 days: Energy for household use (e.g., firewood, charcoal, kerosene, diesel, gas, electricity, candles, battery charging, and other energy sources); water; personal apparel; medicines/drugs (including traditional medicine); local transport (daily travel excluding that for health and education); other nonfood items
- Nonfood consumption expenditures during the last 6 months: clothing and other apparel; home equipment; house rent and repair; health (including traditional medicine); education (including preschool and adult education); travel/trips (overnight travel excluding health and education); other
- Value of assets: household items; agricultural items

The estimation of total expenditures was done based on the daily expenditures after recalculating the various timeframes. The variables used in the calculations and their definitions are described in Table 1.

**Table 1:** Definitions used for creating variables

Out-of-pocket health expenditure	OOPPs refer to the net payment made by households for receiving health care which include the cost of medicines, medical products, outpatient care, dental, travel cost for receiving health care (although it is not included in WHO method, we added traveling cost as one of the burdens to Myanmar people in seeking health care), and inpatient care.
Household consumption expenditure	Household total consumption, either monetary or in-kind payment for all expenditure.
Food expenditure	Total expenditure on all foodstuffs except alcoholic beverages, tobacco, and food consumption outside the home. The local median value at the time of survey used as a reference value of nonpurchased food items.
Rent expenditure	Expenditure on house rent and repaired are included under rent expenditure.
Utility expenditure	Expenditure on energy, including firewood, charcoal, kerosene, diesel, gas (propane or other gases), public electricity, electricity from private sources, candles, and battery charging; and water are included.
Medicines (drugs)	Expenditure on traditional medicines, medicines obtained with vouchers (e.g., prescriptions from doctors or other health professionals), other medicines/drugs (e.g., cold remedies and vitamins), and other health care nondurables (e.g., bandages and birth spacing methods) are included.
Medical products and equipment	Expenditure on medical devices (e.g., eye glasses and hearing aids).
Outpatient care (outpatient)	Expenditure on out-patient care at public hospitals/health centers/clinics, out-patient care at private hospitals/health centers/clinics, home visits by doctors or other health professionals, care from traditional healer and other health care.
Dental	Expenditure on dental care.
Travel	Expenditure on health-related transportation costs.
Inpatient care	Expenditure on in patient stays/long-term care in public hospitals and in patient stay/long-term care in private clinics.
Income	Daily average income to be consistent with expenditure unit.
Children	Number of children under 5 years.
Household size	Household size.

### Analytical approach

To measure financial protection, two main indicators, namely, catastrophic spending and the impoverishing effects of payment for health care, are used in the literature [4].

Two definitions are widely used to estimate catastrophic health care expenditure:

1. The sustainable development goal method: when a household’s OOPPs for receiving health care exceeds certain share of household income or consumption [15].
2. The method proposed by the World Health Organization (WHO): when a household’s OOPPs for health care exceeds certain share of household’s capacity to pay [16].

The impoverishing effects of health care spending was defined as total expenditures falling below the poverty line after paying for receiving health care [17]. In our study, we only focused on catastrophic spending because the choice of the poverty line required for the estimation of impoverishment is sensitive in Myanmar.

To identify households with catastrophic health care spending, we applied the WHO method mentioned above, which is based on a food-based basic need line to estimate household’s capacity to pay. We used the standard WHO approach and the modified WHO approach [16]. The two approaches differ in the calculation of the basic need line and in the predefined thresholds, which leads to differences in the estimated incidence of catastrophic expenditures. Both approaches use OOPPs as the numerator, including both the formal and informal payments made by household for health services. The denominator is the capacity to pay, which is calculated as total household expenditures minus subsistence expenditures, i.e., the expenditures necessary for basic needs. Where food expenditures are regarded as basic needs, then the average spending on food per person by households (food spending between the 45th and 55th percentiles) is used as the standard amount for subsistence expenditures. However, when food expenditures are less than subsistence expenditures, the capacity to pay is defined as total household expenditures minus food expenditures in the WHO standard method. The modified WHO approach, on the other hand, does not require this correction and allows for a negative capacity to pay. Another difference is in the equivalence scales used. The standard WHO equivalence scale is used for the standard WHO approach, and the Organization for Economic Cooperation and Development (OECD) equivalence scale is used for the modified WHO approach [3, 4]. A comparison of estimates of catastrophic health care expenditures using these two approaches can be found in Table 2. Details on the calculation of catastrophic expenditures [16] is presented in Additional file 1.

**Table 2:** Comparison of the two methods used in the estimation of catastrophic health care expenditure

Method	Numerator	Denominator Capacity to pay	Measure of basic needs	Equivalence scale used	Catastrophic expenditure thresholds
Standard WHO approach ^a^	Out-of-pocket-payment	Total household expenditure minus a standard amount representing household basic needs spending based on food if this amount is less than or equal to actual food spending. (or) Total household expenditure minus actual food spending if the amount representing household basic needs spending is greater than the actual food spending.	Average spending on food per (equivalent) person among households whose food spending is between the 45th and 55th percentiles	WHO standard equivalence scale *equivalence size* = *household size* ^0.56^	20%, 30%, 40%
Modified WHO approach ^a^	Out-of-pocket-payment	Total household expenditure minus a standard amount representing household basic needs based on food	Average spending on food per (equivalent) person among households whose food spending is between the 45th and 55th percentiles	OECD equivalence scale *equivalence size*_*=*_ 1 + 0.7*(number of adults − 1) + 0.5*(number of children under 5 years)	20%, 30%, 40%

^a^ Source: (Xu 2005)

### Statistical methods

**Fig. 1:**
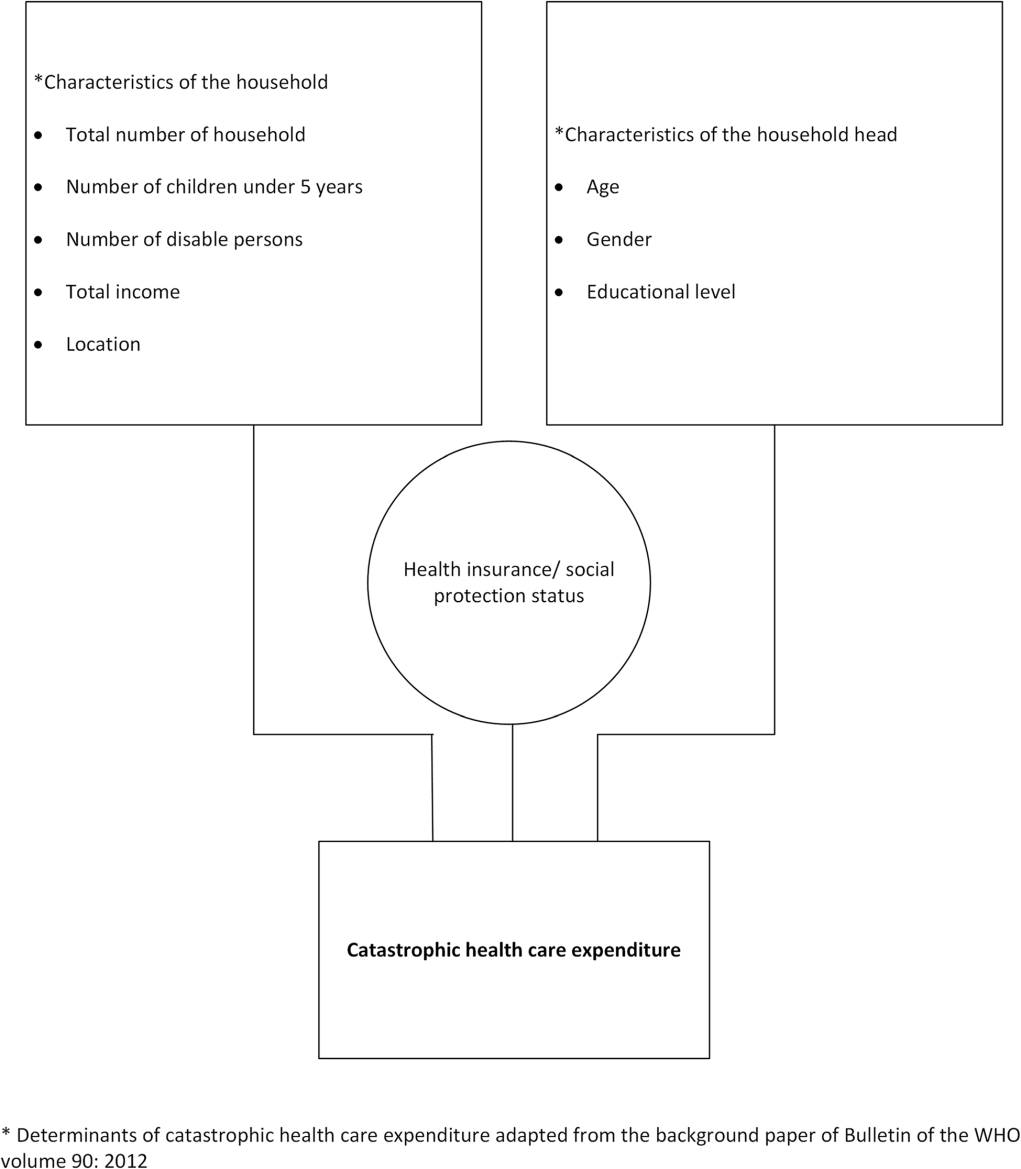
Conceptual framework of determinants of catastrophic health care expenditure

We first calculated the incidence of catastrophic expenditures using the two WHO approaches described above (standard and modified), and three thresholds, namely, 20, 30, and 40% of the household’s capacity to pay. We counted the number of households that exceeded the given thresholds. Results are presented for the total sample in 2013 and 2015. We also present the distribution of catastrophic OOPPs by consumption quintiles: poorest, second, third, fourth, and the richest quintile. In addition, regression analyses were carried out to identify the relation between sociodemographic characteristics and catastrophic health care expenditures at all three thresholds using both approaches. Sociodemographic characteristics included households’ LIFT intervention status; region; age, gender, and education of the household head; total number of household members; number of children under 5 years old; number of disabled persons in the household; and the total monthly household income. These sociodemographic variables are in line with the determinants of catastrophic health care expenditure described in the background paper of the Bulletin of the WHO [18]. The conceptual framework of our analysis is provided in Fig. 1. All analyses were carried out using the software package STATA 14.

## Results

The incidence of catastrophic expenditures for the two approaches at the 20, 30, and 40% threshold levels is shown in Table 3. In 2013, the incidence of catastrophic expenditure was 7% (standard WHO approach) and 41% (modified WHO approach) at the 40% threshold level, while in 2015, it was slightly lower than 6% (standard WHO approach) and 39% (modified WHO approach). The mean OOPPs expenditure per capita per day in 2013 was 526 MMK (approximately 0.6 USD) (standard WHO approach) and 138 MMK (approximately 0.2 USD) (modified WHO approach), while in 2015, it was 1074 MMK (approximately 1 USD) (standard WHO approach) and 247 MMK (approximately 0.2 USD) (modified WHO approach). Thus, in the standard WHO approach, the incidence of catastrophic expenditure was slightly lower in 2015 than in 2013, although mean OOPPs expenditures nearly doubled in 2015.

**Table 3:** Catastrophic health care expenditure, and mean OOPP expenditure (daily per capita) at different levels of threshold by different approaches

	Share of respondents who experienced catastrophic health care expenditure		Mean OOPP expenditure, in MMK / in USD	
	2013	2015	2013 1 USD = 858 MMK	2015 1 USD = 1050 MMK
Equivalized basic needs line, standard WHO approach:				
Catastrophic expenditure (20% threshold)	20.90	17.85	291/0.3	603/0.6
Catastrophic expenditure (30% threshold)	12.50	9.79	385/0.4	855/0.8
Catastrophic expenditure (40% threshold)	7.00	6.35	526/0.6	1074/1.0
Equivalized basic needs line, modified WHO approach:				
Catastrophic expenditure (20% threshold)	47.80	46.78	142/0.2	263/0.3
Catastrophic expenditure (30% threshold)	43.00	41.37	142/0.2	255/0.2
Catastrophic expenditure (40% threshold)	40.60	38.54	138/0.2	247/0.2

The distribution of catastrophic expenditures by income quintiles are shown in Table 4. For both approaches, we found that catastrophic expenditures were lower than or similar to levels when the threshold was increased. Using the standard WHO approach, catastrophic expenditures were highest within the second quintile at 20 and 30% threshold levels, while in the fourth quintile, it was highest at the 40% threshold level in 2013. In 2015, the highest levels of catastrophic expenditures were observed in the richest income quintile at all threshold levels. The modified WHO approach found that catastrophic expenditures were highest in the second quintile in 2013 and the poorest quintile in 2015 at all four threshold levels in both years.

**Table 4:** Share of respondents who experienced catastrophic health care expenditure, in each income quintile

	2013					2015				
	Poorest	2nd	3rd	4th	Richest	Poorest	2nd	3rd	4th	Richest
Equivalized basic needs line, standard WHO approach										
Catastrophic expenditure (20% threshold)	22.50	26.50	18.00	20.00	17.50	17.17	15.88	17.60	18.88	19.74
Catastrophic expenditure (30% threshold)	12.00	14.50	13.00	12.00	11.00	6.01	9.44	11.16	9.87	12.45
Catastrophic expenditure (40% threshold)	5.50	7.0	7.00	8.00	7.50	3.43	6.87	6.44	6.44	8.58
Equivalized basic needs line, modified WHO approach										
Catastrophic expenditure (20% threshold)	73.00	78.00	45.50	25.00	17.50	78.97	77.68	35.19	21.46	20.60
Catastrophic expenditure (30% threshold)	73.00	78.00	34.50	17.00	12.50	78.97	76.39	25.75	12.88	12.88
Catastrophic expenditure (40% threshold)	73.00	78.00	31.00	12.50	8.50	78.97	75.97	18.88	9.44	9.44

The average levels of OOPPs in each income quintile are shown in Table 5. Using the standard WHO approach, there was a six times difference in the average OOPPs value between the poorest and richest quintiles in 2013 (0.2 USD vs. 1.2 USD per capita per day at the 40% threshold level). In 2015, this difference was 10 times greater in the richest quintile (0.2 USD vs. 2.1 USD per capita per day). Using the modified WHO approach, the average OOPPs in the richest quintile was 12 times higher than the poorest quintile in 2013 and 20 times higher in 2015 at the 40% threshold level.

**Table 5:** Mean level of OOPPs household expenditure (daily per capita) in each income quintile

	2013 MMK/USD (1 USD = 858 MMK)					2015 MMK/USD (1 USD = 1050 MMK)				
	Poorest	2nd	3rd	4th	Richest	Poorest	2nd	3rd	4th	Richest
Equivalized basic needs line, standard WHO approach										
Catastrophic expenditure (20% threshold)	124/0.1	145/0.2	201/0.2	393/0.5	699/0.8	142/0.1	316/0.3	396/0.4	658/0.6	1367/1.3
Catastrophic expenditure (30% threshold)	163/0.2	199/0.2	238/0.3	534/0.6	884/1.0	174/0.2	442/0.4	503/0.5	924/0.9	1758/1.7
Catastrophic expenditure (40% threshold)	213/0.2	245/0.3	293/0.3	691/0.8	1058/1.2	186/0.2	504/0.5	590/0.6	1164/1.1	2180/2.1
Equivalized basic needs line, modified WHO approach										
Catastrophic expenditure (20% threshold)	48/0.1	65/0.1	101/0.1	340/0.4	699/0.8	54/0.1	98/0.1	257/0.2	615/0.6	1326/1.3
Catastrophic expenditure (30% threshold)	48/0.1	65/0.1	121/0.1	438/0.5	829/1.0	54/0.1	100/0.1	312/0.3	811/0.8	1735/1.7
Catastrophic expenditure (40% threshold)	48/0.1	65/0.1	128/0.1	536/0.6	1032/1.2	54/0.1	100/0.1	369/0.4	960/0.9	2082/2.0

The percentage of daily health expenditures per capita in each income quintile that was spent on each service (i.e., pharmaceuticals, medical products, outpatient care, dental, travel, and inpatient care) based on the two WHO approaches are shown in Tables 6 and 7. Using both approaches, we found that OOPPs for pharmaceuticals, outpatient, and inpatient care were the three main areas of payments for services among all income quintiles in both years. In all income quintiles in 2013, the highest share of OOPPs were spent on pharmaceuticals (38–49% using the standard WHO approach, 47–56% using the modified WHO approach). In 2015, the poorest quintile (68%) and the third quintile (42%) spent most on outpatient care while the other three income quintiles spent most (55–59%) on inpatient care based on estimates calculated using the standard WHO approach. The modified WHO approach shows that the poorest quintile spent approximately 80% of OOPPs on medicines and outpatient care in both years. In 2015, the fourth and richest quintiles spent most OOPPs on inpatient care.

**Table 6:** Share of OOPPs in each income quintile that is spent on each service: equivalized basic needs line, standard WHO approach (Partial normative food spending method)

Percentage of each income quintile that is spent on each service		2013						2015					
		Medicines	Medical products	Outpatient care	Dental	Travel	Inpatient care	Medicines	Medical products	Outpatient care	Dental	Travel	Inpatient care
Catastrophic expenditure (20% threshold)	Poorest	52.60	1.41	20.32	0.00	2.36	23.32	35.12	0.14	43.43	0.21	1.62	19.47
	2nd	51.26	0.00	29.36	0.14	1.62	17.63	21.54	0.38	31.49	0.00	2.11	44.49
	3rd	46.04	0.00	22.80	1.90	1.82	27.44	22.02	0.90	38.48	0.00	6.07	32.53
	4th	48.28	0.02	38.06	0.00	1.96	11.69	25.91	3.90	28.76	0.06	4.38	37.00
	Richest	54.13	0.11	19.56	0.00	1.65	24.55	27.07	0.00	21.43	0.01	3.65	47.84
Catastrophic expenditure (30% threshold)	Poorest	46.46	2.02	19.95	0.00	2.20	29.37	16.16	0.00	57.04	0.00	1.17	25.64
	2nd	50.86	0.00	25.56	0.19	1.95	21.44	18.34	0.00	31.20	0.00	2.19	48.26
	3rd	45.01	0.00	19.26	2.22	1.46	32.06	17.30	1.12	39.19	0.00	5.99	36.40
	4th	48.17	0.01	37.43	0.00	0.82	13.58	24.49	4.83	23.98	0.00	3.39	43.32
	Richest	52.21	0.14	17.00	0.00	1.90	28.75	26.51	0.00	16.56	0.00	3.41	53.52
Catastrophic expenditure (40% threshold)	Poorest	38.19	3.35	27.71	0.00	2.88	27.86	7.86	0.00	67.67	0.00	1.75	22.73
	2nd	42.95	0.00	22.43	0.00	0.95	33.67	12.11	0.00	30.54	0.00	2.53	54.82
	3rd	36.40	0.00	20.98	3.35	2.05	37.21	21.02	0.10	41.68	0.00	2.49	34.72
	4th	40.95	0.01	42.35	0.00	0.95	15.74	24.64	5.24	18.61	0.00	2.96	48.54
	Richest	48.94	0.17	16.49	0.00	2.33	32.07	24.92	0.00	13.47	0.00	2.73	58.89

**Table 7:** Share of OOPPs in each income quintile that is spent on each service: equivalized basic needs line, modified WHO approach (continued)

Percentage of each income quintile that is spent on each service		2013						2015					
		Medicines	Medical products	Outpatient care	Dental	Travel	Inpatient care	Medicines	Medical products	Outpatient care	Dental	Travel	Inpatient care
Catastrophic expenditure (20% threshold)	Poorest	55.05	1.44	22.27	0.00	2.16	10.08	45.54	0.97	39.37	0.12	1.56	12.43
	2nd	55.94	0.07	28.24	0.11	2.00	13.64	32.94	0.46	34.51	0.01	2.34	29.73
	3rd	50.70	0.00	23.14	1.57	1.61	22.78	26.47	0.69	36.87	1.11	6.54	28.33
	4th	50.27	0.02	36.83	0.00	1.86	11.02	26.39	3.67	29.15	0.06	4.82	35.91
	Richest	54.13	0.11	19.56	0.00	1.65	24.55	27.76	0.00	21.23	0.01	3.74	47.27
Catastrophic expenditure (30% threshold)	Poorest	55.05	1.44	22.27	0.00	2.16	19.08	45.53	0.97	39.37	0.12	1.56	12.43
	2nd	55.94	0.07	28.24	0.11	2.00	13.64	32.98	0.47	34.35	0.01	2.33	29.86
	3rd	49.55	0.00	22.85	0.17	2.77	24.10	24.50	0.78	36.26	0.95	6.40	31.11
	4th	47.95	0.01	39.09	0.00	1.28	11.67	24.73	4.61	26.89	0.07	3.86	39.83
	Richest	54.00	0.13	16.47	0.00	1.78	27.62	26.33	0.00	17.54	0.00	3.73	52.40
Catastrophic expenditure (40% threshold)	Poorest	55.05	1.44	22.27	0.00	2.16	19.08	45.54	0.97	39.37	0.12	1.56	12.43
	2nd	55.94	0.07	28.24	0.11	2.00	13.64	32.91	0.47	34.29	0.01	2.34	29.98
	3rd	49.87	0.00	21.47	1.81	1.65	25.21	22.63	0.90	37.22	0.85	5.92	32.48
	4th	47.21	0.01	38.62	0.00	1.18	12.99	24.03	4.86	24.51	0.00	3.33	43.27
	Richest	55.91	0.16	14.92	0.00	2.11	26.90	27.43	0.00	13.12	0.00	2.60	56.85

The data on catastrophic health care expenditures were further analyzed by logistic regression analysis to determine the association between sociodemographic characteristics and catastrophic health care expenditures at the three threshold levels using both approaches for data from 2013 and 2015.

Geographical location, gender and education of the household head, total number of household members, number of children under 5, and number of disabled persons in the household were statistically significantly associated with catastrophic health care expenditures in 2013. In 2013, the geographical location of households was significantly associated with catastrophic health care expenditures at the 20% (OR = 0.57) and 30% (OR = 0.51) thresholds using the WHO standard approach and among all three thresholds levels using the WHO modified approach (20% OR = 0.45, 30% OR = 0.45, and 40% OR = 0.46. In other words, people living in the Hilly zone were approximately 50% less likely to face catastrophic expenditures than those living in the Delta zone. Female-headed households were 1.02 times more likely to face catastrophic expenditures at 20 and 30% threshold levels using the WHO standard method. Higher levels of education of the household head lowered the chance of catastrophic expenditure at the 20% threshold level (OR = 0.82) using the WHO standard approach, and at the 30% (OR = 0.83) and 40% (OR = 0.81) threshold levels using the WHO modified approach. Larger household sizes were associated with greater likelihood of facing catastrophic expenditures at all three threshold levels (OR = 1.63, 1.78, 1.85) using the WHO modified approach. Having more children in the family under 5 years old was associated with a greater the likelihood of catastrophic expenditures, with odds ratios ranging from 1.3 to 1.7 times at the 20 and 30% threshold levels using the WHO standard approach and the 30 and 40% threshold levels using the WHO modified approach. The number of disabled persons living in the household also increased the chance of catastrophic expenditures by 1.78 to 1.73 times and 1.68 to 1.70 times at the 20 and 30% threshold levels using the WHO standard and modified approaches, respectively. Detailed results are provided in Table 8.

**Table 8:** Relationship between sociodemographic factors and catastrophic health care expenditure (binary logistic regression with the 2013 data)

Independent variables	Catastrophic Expenditure (Yes = 1, No = 0) *N* = 999																	
	WHO standard 20%			WHO standard 30%			WHO standard 40%			WHO modified 20%			WHO modified 30%			WHO modified 40%		
	OR	95% CI		OR	95% CI		OR	95% CI		OR	95% CI		OR	95% CI		OR	95% CI	
LIFT Intervention	0.7995	0.5581	1.1452	0.7689	0.4985	1.1858	0.7711	0.4458	1.3338	0.9467	0.6880	1.3026	0.8855	0.6358	1.2333	0.8512	0.6066	1.1944
Hilly Region	0.5706	0.3767	0.8643	0.5069	0.2972	0.8645	0.6359	0.3202	1.2630	0.4469	0.3141	0.6360	0.4545	0.3138	0.6583	0.4630	0.3160	0.6783
Dry Zone	1.1346	0.7748	1.6614	1.2559	0.7950	1.9839	1.4228	0.7913	2.5583	1.0141	0.7186	1.4310	1.0596	0.7418	1.5135	1.1096	0.7703	1.5984
Age of household head	0.9811	0.6248	1.5405	0.7283	0.4147	1.2790	0.7873	0.3898	1.5903	1.3028	0.8678	1.9560	1.2062	0.7882	1.8458	1.2401	0.8012	1.9194
Gender of household head	1.0203	1.0075	1.0333	1.0256	1.0100	1.0414	1.0157	0.9963	1.0355	1.0085	0.9972	1.0199	1.0080	0.9962	1.0200	1.0099	0.9978	1.0222
Education of household head	0.8192	0.6751	0.9941	0.8643	0.6792	1.0998	0.8090	0.5905	1.1087	0.8512	0.7212	1.0046	0.8251	0.6936	0.9815	0.8129	0.6798	0.9721
Total number of households	1.0553	0.9674	1.1513	1.0255	0.9190	1.1445	0.9881	0.8580	1.1380	1.6255	1.4820	1.7828	1.7751	1.6089	1.9586	1.8542	1.6747	2.0529
Number of under 5 children in the household	1.7365	1.3270	2.2723	1.6296	1.1700	2.2698	1.3132	0.8496	2.0295	1.2567	0.9765	1.6173	1.3257	1.0232	1.7178	1.3787	1.0594	1.7941
Number of disable person in the household	1.7860	1.1608	2.2723	1.7324	1.0733	2.7965	1.5112	0.8465	2.6978	1.6834	1.0494	2.7004	1.6955	1.0497	2.7387	1.3293	0.8236	2.1456
Total household income	0.9827	0.9537	1.0125	0.8924	0.7923	1.0051	0.9464	0.8292	1.0802	0.9916	0.9713	1.0124	0.9946	0.9736	1.0161	0.9972	0.9762	1.0186
Constant	0.1124	0.0415	0.3050	0.0981	0.0280	0.3438	0.0860	0.0175	0.4237	0.0786	0.0319	0.1936	0.0484	0.0188	0.1247	0.0313	0.0118	0.830
LR chi^2^	60.65			48.64			18.59			213.38			267.87			295.48		
Prob > chi^2^	0.0000			0.0000			0.0458			0.0000			0.0000			0.0000		
Pseudo R^2^	0.0592			0.0646			0.0367			0.1543			0.1962			0.2189		

Geographical location, gender of the household head, total number of the household members, the number of children under 5, the number of disabled persons in the household, and total household income were significantly associated with catastrophic health care expenditures in 2015. Using the WHO modified approach, households living in the hilly region were nearly two times (OR = 1.65, 1.43, 1.53) more likely to have catastrophic expenditures at the 20, 30, and 40% threshold levels. Female-headed households had a 1–2% greater likelihood of catastrophic expenditures at the 20 and 30% threshold levels using the WHO standard method. Larger households were associated with a 1.85, 2.12, and 2.22 times greater likelihood of having catastrophic expenditures at the 20, 30, and 40% threshold levels using the WHO modified approach. Having more children under 5 was associated with a 37% increased likelihood of having catastrophic expenditures at the 20% threshold level using the WHO standard approach. Having disabled persons in the household increased the odds of catastrophic payments by 1.76, 1.79, and 1.77 at 20, 30, and 40% threshold levels using the WHO standard approach, and by 2.21, 1.91, and 1.93 using the WHO modified approach. Finally, higher household incomes were associated with a reduced likelihood of catastrophic expenditures, with OR = 0.85, 0.82, and 0.81 at 20, 30, and 40% threshold levels using the WHO modified approach. Detailed results are provided in Table 9.

**Table 9:** Relationship between sociodemographic factors and catastrophic health care expenditure (binary logistic regression with the 2015 data)

Independent variables	Catastrophic Expenditure (Yes = 1, No = 0) *N* = 1163																	
	WHO standard 20%			WHO standard 30%			WHO standard 40%			WHO modified 20%			WHO modified 30%			WHO modified 40%		
	OR	95% CI		OR	95% CI		OR	95% CI		OR	95% CI		OR	95% CI		OR	95% CI	
LIFT Intervention	1.0587	0.7233	1.5497	1.0305	0.6355	1.6709	1.012	0.5658	1.8095	0.8674	0.6267	1.2005	0.8393	0.5983	1.1773	0.8325	0.5882	1.1783
Hilly Region	1.3576	0.9151	2.0142	0.9701	0.5892	1.5970	0.8204	0.4514	1.4910	1.6515	1.1758	2.3198	1.4318	1.0052	2.0394	1.5335	1.0660	2.2062
Dry Zone	1.0071	0.6672	1.5203	0.8047	0.4826	1.3417	0.6777	0.3643	1.2608	1.0796	0.7630	1.5275	0.9474	0.6566	1.3668	0.9642	0.6592	1.4105
Age of household head	0.7831	0.5076	1.2082	0.9264	0.5447	1.5754	1.2252	0.6575	2.2831	0.8279	0.5648	1.2136	0.8568	0.5700	1.2880	0.9629	0.6319	1.4673
Gender of household head	1.0133	1.0001	1.0258	1.0158	1.000	1.0317	1.0126	0.9937	1.0318	1.0104	0.9991	1.0218	1.0043	0.9926	1.0161	0.9994	0.9875	1.0115
Education of household head	1.1261	0.9351	1.3560	1.0136	0.7946	1.2929	1.0414	0.7740	1.4012	1.0148	0.8604	1.1970	0.9615	0.8082	1.1438	0.9441	0.7892	1.1293
Total number of households	0.9477	0.8639	1.0398	0.9451	0.8398	1.0635	0.9559	0.8291	1.1020	1.8497	1.6713	2.0475	2.1213	1.9002	2.3683	2.2228	1.9835	2.4910
Number of under 5 children in the household	1.3664	1.0491	1.7795	1.1700	0.8247	1.6600	1.3463	0.8985	2.0173	1.1771	0.9165	1.5117	1.0661	0.8283	1.3721	1.0956	0.8492	1.4134
Number of disable person in the household	1.7598	1.3201	2.3456	1.7909	1.2862	2.4935	1.7687	1.1972	2.6131	2.2092	1.5636	3.1212	1.9125	1.3645	2.6806	1.9276	1.3703	2.7115
Total household income	0.9533	0.8951	1.0153	0.9894	0.9152	1.0697	0.9690	0.8783	1.0689	0.8486	0.8018	0.8980	0.8165	0.7676	0.8685	0.8151	0.7647	0.8688
Constant	0.1255	0.0453	0.3472	0.0610	0.0167	0.2231	0.0270	0.0073	0.1606	0.7978	0.0315	0.2018	0.0684	0.0260	0.1796	0.0524	0.0194	0.1413
LR chi^2^	35.28			21.05			15.75			332.04			394.20			422.14		
Prob > chi^2^	0.0001			0.0207			0.1070			0.0000			0.0000			0.0000		
Pseudo R^2^	0.0323			0.0282			0.0286			0.2066			0.2499			0.2723		

## Discussion

Using the standard WHO approach, our findings show that the incidence of catastrophic health care expenditure ranged from 7 to 21% in 2013 and from 6 to 18% in 2015, depending on the threshold used. This incidence is lower than what was observed in previous Myanmar studies conducted in Magway region (23.6%), selected states and regions (32.9%), and among poor families living in two mountainous states (26%), all of which applied the 40% threshold and the WHO standard approach [8, 10, 11]. These differences may be observed because the studies were conducted across different geographic locations. Additionally, access to high cost facilities varied across the studies, since such facilities are only located in large cities. The incidence of catastrophic expenditures in 2013 found in our study were similar to that observed in the Philippines (7.7%) in the same year using the same WHO standard approach and the same threshold level of 40% [19]. In Indonesia and Thailand in 2013, the incidence of catastrophic health care expenditures was much lower (4.4 and 2.3%, respectively) based on the Sustainable Development Goal approach. This approach considers health care expenditures to be those that exceed 10% of the household’s total expenditure on health care [20, 21].

Overall, our findings show that in Myanmar, the incidence of catastrophic health care expenditure was lower in 2015 compared to that in 2013, although the percent contribution of OOPPs to the total health expenditure was higher in 2015 (74%) than in 2013 (64%) [5]. This finding contradicts what was observed in a previous study by Xu, Evans et al. (2003), which found a positive relationship between the proportion of households with catastrophic health expenditures and the share of OOPPs in total health expenditures. The reasons for this trend may be that household incomes increased during those years (a decline in poverty is observed over the period 2004–2015), or it could be because poor households were more likely to forgo treatment [3, 22]. The issue of forgoing treatments was not captured by a catastrophic expenditure analysis.

Our study also found that the mean OOPPs expenditure per capita per day in 2015 was nearly double that observed in 2013. There was 1.2 times inflation of the exchange rate between the two studied years, but alone this trend does not explain the increase in mean OOPPs. Rather, this may have been due to increases in health care costs other than inflation since mean OOPPs expenditures increased in USD value at this time as well. Alternatively, it could be due to increased household wealth. In addition, country level data show that OOPPs per capita was increased from 15 USD in 2013 to 44 USD in 2015,although the percent contribution of OOPPs to total health expenditures decreased [5]. This also suggests that health care costs were increasing. However, it should be noted that other potential causes may also exist, such as a reduction in free health care provided through public services and changes in the incidence of diseases towards more chronic illnesses.

Analyses of the distribution of catastrophic health care expenditures across income quintiles found that the share of catastrophic expenditures among the poorest households decreased with increasing thresholds in both studied years. Moreover, the share of catastrophic expenditures in the poorest quintile was at the lowest across all income quintiles at the 40% threshold using the WHO standard approach. This finding was similar to that reported in a study conducted in China and another study conducted in 8 countries (Bangladesh, Bhutan, India, Maldives, Nepal, Sri Lanka, Thailand, and Temor-Leste). However, it differs from trends observed in Thailand, where the share of catastrophic OOPPs did not vary by income quintile [18, 23]. In contrast, the highest proportion of catastrophic expenditures were observed among the lowest income group in Georgia and Nigeria [24, 25]. Our finding of lower incidences of catastrophic health care expenditures and lower levels of mean OOPPs in the poorest quintile is in line with prior findings of a lower level of health care expenditure in lower income countries and poor households [6]. Households facing high health care costs might forgo receiving care because of unaffordable charges. However, this issue requires further investigation because we were not able to examine the reason for the lower incidence of catastrophic health expenditures observed among poor households in Myanmar.

We found that pharmaceutical costs are the highest spending area for both the poorest and the richest quintile using the WHO standard approach and across all income quintiles using on the WHO modified approach. This finding is consistent with a study of eight countries in the WHO South-East Asia Region, which found that the proportion of total OOPPs attributable to pharmaceutical costs is 34.05–81.89% [23]. One study in the Philippines also found that pharmaceutical costs are the main driver of health spending and is as high as two-thirds of the total health spending [19]. Wegner, Graves et al. 2011 also found that between 41 and 56% of households from low- and middle income countries make major OOPPs for pharmaceuticals [6]. The financial burden due to pharmaceutical cost is not only found in countries that lack a prepayment system. But also in countries with a well-established health insurance system that require OOPPs for most pharmaceuticals [26, 27]. To control the higher spending on pharmaceuticals, it is important to consider the expansion of pharmaceutical coverage policies, the proper evaluation of essential drug policy on access, use, and affordability, promotion of standard quality generic drugs and active purchasing strategies, and monitoring of medicines utilization [6, 28].

Sociodemographic factors associated with catastrophic expenditures include: geographic location; gender and education of household head; household size, number of children under 5 years old and disabled persons in the household; and total household income. These findings are consistent with a study conducted in China showing that female-headed households, those with low education, and households with elderly or chronically ill family members are more likely to experience catastrophic health expenditures [18]. Similarly, risk factors associated with catastrophic health expenditure found in sub-Saharan African countries include household economic status, type of health care provider, socio-demographic characteristics of household members, type of illness, social insurance schemes, geographical location and household size [29, 30]. One study in India found that the type of village is correlated with catastrophic health care expenditure [31]. This study additionally found that households without insurance faced a greater risk of catastrophic expenditures. However, our study did not include information on the health insurance status of participants because it was not available. Additionally, only a small proportion of the population in Myanmar possesses health insurance.

We found that the incidence of catastrophic expenditures varied by the approach used to estimate expenditures. These variations emphasize the importance of being consistent in catastrophic health care expenditure analyses. Specifically, our study applied two different approaches, which yielded considerably different incidence rates. The standard WHO approach controls for negative results in calculating capacity to pay. Therefore, it produces a lower incidence of catastrophic expenditures than the modified WHO approach. However, the standard WHO approach better reflects the real-world situation because it uses the actual food-spending costs if the representing household basic needs spending is greater than the actual food spending. The choice of the threshold in a catastrophic expenditure study is also important. As shown by our results, the lower threshold level of 20% of capacity to pay captures up to three times more catastrophic expenditure cases than those captured at the 40% threshold level. There are also other approaches for estimating catastrophic health care expenditures. For example, the WHO European regional office considers not only food but also rent and utilities spending as a basic need [32]. However, this approach is not recommended for lower-income countries because the inclusion of more spending categories may cause a negative result in calculating the capacity to pay, leading to an overestimation of catastrophic expenditures. This confirms that a catastrophic expenditure study should be carefully designed to provide meaningful evidence for policy.

Our study has some limitations. First, data were only available for the LIFT intervention area for two rounds of the LIFT survey, which means that these are subnational data and also that they cannot be used to study trends. Therefore, the results are not nationally representative and can not be used for causal inferences. Second, no information about the insurance or social protection status of the studied population was available, which could have been an important explanatory variables as suggested in other studies [18, 30, 31]. Third, most of the expenditure data retrieved from the LIFT survey capture daily expenditures, and therefore we had to also transform other data (such as income) into daily expenditures. Lastly, the LIFT intervention area is rural, which means that health care utilization patterns and costs might be different from those in urban areas. More studies are needed to collect and analyze catastrophic health care expenditure data at the national level.

## Conclusion

Our study findings provide a base for key policy conclusions as the magnitude of the catastrophic health expenditure is an indicator of the effectiveness of the current health financing arrangements. Although the level of catastrophic health care expenditure varies depending on the approach and threshold used, the problem of catastrophic expenditures in Myanmar cannot be denied. The government of Myanmar needs to scale up the current SSS or establish a new financial protection mechanism for the population. Vulnerable groups, such as households with a low educated household head, households with children under the age of 5 years or disabled persons, and low-income households should be a priority in the improvement of access to essential health care. The incidence of catastrophic expenditures that we captured in our study, might only be the tip of the iceberg as our data only represent subnational trends. Additionally, the uneven distribution of catastrophic expenditures across wealth quintiles may also show another major problem: individuals not seeking needed care because of OOPPs. This problem is not visible in catastrophic health care expenditure research and requires a separate investigation.

## Additional file

Additional file 1: Calculation of catastrophic health expenditure. (DOCX 23 kb)

## Data Availability

The datasets generated during and analyzed during the current study are available from the corresponding author on reasonable request.

## Abbreviations

ASEAN: Association of Southeast Asian Nations
LIFT: Livelihoods and Food Security Trust Fund
MMK: Myanmar Kyat
OECD: Organization for Economic Cooperation and Development
OOPPs: Out-of-pocket payments
SSS: The Social Security Scheme
USD: Unites State Dollar
WHO: World Health Organization
