# Supplementary material for: Catastrophic health care expenditure in Myanmar: policy implications in leading progress towards universal health coverage
Source: Int J Equity Health. 2019 Jul 30;18:118. doi: 10.1186/s12939-019-1018-y (PMC6664746; doi:10.1186/s12939-019-1018-y)
Supplement: Supplementary file 1 — Calculation of catastrophic health expenditure. (DOCX 23 kb) [file 12939_2019_1018_MOESM1_ESM.docx]

Additional file 1

Calculation of catastrophic health expenditure

| Considering the economy scale of household consumption, the household equivalence scale is used rather than actual household size. The equivalence scale is:  *eqsize _h_* = *hhsize _h_^β^*  where *hhsize_h_* is the household size. The value of the parameter *β* has been estimated from previous studies based on 59 countries’ household survey data, and it equals 0.56.  Subsistence spending can be calculated as follows:  1. Generate the food expenditure share (*foodexp_h_*) for each household by dividing the household’s food expenditure by its total expenditure   \| *foodexp_h_* \| = \| *food_h_* \| \| --- \| --- \| --- \| \| *exp_h_* \|     2. Generate the equivalent household size for each household as  *eqsize_h_* = *hhsize _h_*^0.56^  3. Divide each household food expenditure by the equivalent household size to get equivalised food expenditures (*eqfood_h_*)   \| *eqfood_h_* \| = \| *food_h_* \| \| --- \| --- \| --- \| \| *eqsize_h_* \|   4. Identify the food expenditure shares of total household expenditure that are at the 45^th^ and 55th percentile across the whole sample, name these two variables as food45 and food55. If the survey includes a household weighting variable, the percentile calculation should consider the weight.  5. Calculate the weighted average of food expenditure in the 45th to 55th percentile range. This gives the subsistence expenditure per (equivalent) capita, which is also the poverty line (*pl*)   \| *pl* \| = \| Ʃ *w_h_* eqfood_h_* \| \| --- \| --- \| --- \| \| Ʃ *w_h_* \|   where food45<foodexph<food55  6. Lastly, calculate the subsistence expenditure for each household (se*_h_*) as  se_h_ = *pl * eqsize_h_*  A household is regarded as poor (*poor_h_*) when its total household expenditure is smaller than its subsistence spending.  *poor_h_* =1 if exp_h_ < se_h_  *poor_h_* = 0 if exp_h_ ≥ she   1. Out-of-pocket health payments share of household capacity to pay (oopctp)   The burden of health payments is defined as the out-of-pocket payments as a percentage of a household’s capacity to pay.   \| *oopctp_h_* \| = \| *oop_h_* \| \| --- \| --- \| --- \| \| *ctp_h_* \|  1. Catastrophic health expenditure (cata)   Catastrophic heath expenditure occurs when a household’s total out-of-pocket health payments equal or exceed 40% of household’s capacity to pay or non-subsistence spending.  The variable on catastrophic health expenditure is constructed as a dummy variable with value 1 indicating a household with catastrophic expenditure, and 0 without catastrophic  expenditure.  *cata_h_* = 1 if *oop_h_*  *ctp_h_*  ≥ 0.4  *cata_h_* = 0 if *oop_h_*  *ctp_h_*  ˂ 0.4   1. Expenditure quintile (quintile)   The expenditure quintile is ranked by equivalized per capita household expenditure (*eqexp_h_*).    *eqexp_h_* = exp*_h_*  *eqsize*_h_    Note: household weight should be considered when grouping the population by quintile. |
| --- | --- | --- | --- | --- | --- | --- | --- | --- | --- | --- | --- | --- | --- | --- | --- | --- |

*WHO method adapted from Xu.K “Distribution of health payments and catastrophic expenditures Methodology”*
